# *Candida albicans* and Oral Carcinogenesis. A Brief Review

**DOI:** 10.3390/jof7060476

**Published:** 2021-06-12

**Authors:** Michele Di Cosola, Angela Pia Cazzolla, Ioannis Alexandros Charitos, Andrea Ballini, Francesco Inchingolo, Luigi Santacroce

**Affiliations:** 1 Department of Clinical and Experimental Medicine, Università degli Studi di Foggia, 71122 Foggia, Italy; michele.dicosola@unifg.it (M.D.C.); elicio@inwind.it (A.P.C.); 2 Department of Emergency and Urgency, National Poisoning Centre, Riuniti University Hospital of Foggia, 71122 Foggia, Italy; 3 Department of Biosciences, Biotechnologies and Biopharmaceutics, Campus Universitario Ernesto Quagliariello, University of Bari “Aldo Moro”, 70125 Bari, Italy; 4 Department of Interdisciplinary Medicine, University of Bari “Aldo Moro” School of Medicine, 70124 Bari, Italy; francesco.inchingolo@uniba.it (F.I.); luigi.santacroce@uniba.it (L.S.)

**Keywords:** oral infections, *Candida albicans*, oncogenesis, oral cancer, inflammation, oral microbiota

## Abstract

Current medical knowledge and research on patients’ management are still evolving, and several protocols on minimizing risk of infection by *Candida* spp. among the population have developed. The aim of this work is to review the epidemiological and biomolecular characteristics and the various histopathological carcinogenesis hypothesis mechanisms that can occur during *Candida albicans* infections. Current evidence from the literature on the role of *C. albicans* during potentially malignant oral disorders and oral cancer has been sought. Thus, these biomolecular processes can give or contribute to benign lesions, also in precancerous or cancerous situations. Alongside this, the physiological microorganism oral flora (microbiota) can play a crucial role in maintaining oral health during those infections and therefore avoid carcinogenesis.

## 1. Introduction

Pre-cancerous and cancerous oral lesions are among the most common forms of cancer, mainly in developed countries with a male prevalence mostly around the fifth and sixth decades of life [1]. Some of these lesions are already described in ancient times, such as in the “*Iπποκρατικό σώμα*” (*Corpus Hippocraticum*) and later in the manuscripts of the doctors of the Byzantine Roman Empire. Oral cancer, such as squamous cell carcinoma, in more than 50% of cases is preceded by potentially malignant lesions [2,3]. In fact, a characteristic aspect is the presence of white areas in the white and/or red patches (leukoplakia/erythroplakia) on the mucosa, which subsequently evolve into ulcers and/or a lump. The 5-year survival is less than 50% and despite the progress made in terms of early diagnosis and treatment in the last twenty years, it has not significantly improved [2,4]. Potentially malignant lesions, which the WHO defines as precancerous lesions, include leukoplakia, oral lichen planus, and also oral submucosal fibrosis (it is present in Eastern populations where it is customary to chew quid leaf) [5]. These precancerous forms for a differential diagnosis of exclusion from other oral pathologies will be based on the medical history, physical examination, and/or histological examination. In fact, for a pseudo-membranous candidiasis lesion, the main diagnostic criterion will be the clinical objective examination, and instead, for a chronic hyperplastic candidiasis, it will be the histological examination that makes the differential diagnosis from oral leukoplakia [2,6]. Instead, in North America and Europe, high risk human papillomavirus (HPV) infections are responsible for a growing percentage of oropharyngeal cancers among young people; for other infectious agents, this link is still debated (e.g., it has been reported that different species belonging to the *Candida* spp. (mostly *Candida albicans*) that produce endogenous nitrosamines from dietary nitrites are present in the oral cavity, especially in saliva) [7,8].

The *Candida* genus can cause a wide range of pathologies of varying degrees depending on the pathogen and the host’s immune condition. The colonization of the mucous membranes can occur by a change in the microbial population of the microbiota with a preponderant growth of *Candida*, which can then develop into a disseminated form, in synergy to other oral diseases [9,10]. The most frequently encountered forms are superficial infections affecting the skin (intertrigo) and mucous membranes of the female genital system (vaginitis) and oral cavity (thrush). More rarely, there are deep infections resulting from dissemination that lead to a septic state, that can then lead to multi-organ failure (MOF) [11]. Indeed, invasive candidiasis is a systemic infection that includes two main clinical conditions, *Candida* bloodstream infection and deep-seated candidiasis, which lead to a severe septic state. Host risk factors associated with candidemia and invasive candidiasis are mainly related to the state of health, such as immune compromission (neutropenia) and/or environment reasons (such as the hospital one). Therefore, prolonged hospitalization in the ICU, the use of broad-spectrum antibiotics, chemotherapy, mucosal colonization, vascular catheters, parenteral nutrition, major surgery (especially that of the gastrointestinal tract), and renal failure (e.g., hemodialysis) have been demonstrated as important risk factors in multivariate analyses [11,12,13]. In addition, some patients have, already upon admission, a higher risk of developing candidemia due to their underlying medical conditions, such as transplant recipients, diabetics, premature newborns, and elderly patients. Candidiasis and candidemia can have an endogenous or exogenous origin. The endogenous origin is a consequence of the increased colonization of *Candida* spp. of the mucous membranes mainly from the gastrointestinal tract, favored by prolonged exposure to broad-spectrum antibiotics that suppress the natural bacterial flora and increase the growth rate of endogenous *Candida* on the mucous membranes [13,14]. The translocation of the fungus, which also occurs on intact mucous membranes, is favored in the event of damage to the integrity of the mucosa resulting from surgery or chemotherapy. The exogenous origin is favored by solutions of the continuity of the barrier that are realized in the presence of vascular catheters or for prolonged hospitalization, which increase the possibility of infection due to contact with healthcare personnel or the environment itself [14,15].

## 2. The Main Features of *Candida albicans*

### 2.1. Cell Wall Structure and Virulence Factors

*Candida albicans* is a saprophytic fungus belonging to the *Saccharomycetaceae* family found in the human microbiota of the gastrointestinal tract, oral cavity, and vagina [16,17]. The cell wall is a dynamic and fluid structure that constantly changes its composition. It is mainly composed of polysaccharides (phosphorylated mannans, glucans, and chitins), polypeptides, and proteins. The mannan residues are bound to the cell wall via O- and *N*-glycosylation and the structure of these can vary if conditions change in pH and temperature. β-Glucans are in the wall but more internally than the mannans. Lipids’ cell walls mainly are represented by phospholipids and sterols, and by ergosterol [17,18]. These lipids provide the site of action for the synthesis of enzymes involved in the development of the structure/shape of the cell wall (morphogenesis) and are the target of many antifungals. A lipid alteration can occur during the transformation from yeast to mycelium [17,18,19]. The polypeptides and proteins, by binding to the polysaccharides of the cell wall, create many inter and intra species differences regarding hydrophobic properties, adhesion ability (to host cells or other surfaces), and antigenic structure [16,17]. Therefore, the expression of the macromolecular cell wall components also varies within the same cell in order to give them different functions in different parts. Entry into the host cell then occurs mainly with adhesins (ALS1, ALS5, HWP1, and INT1 are the most important), membrane proteins which bind with fibrinogen or laminins of the extracellular matrix of the cell membrane. The adhesion to the cell surface occurs with electrostatic charges and Van der Waals forces [18]. The hydrophobicity of the fungal cell membrane contributes in an important way to the adhesion to inert substrates and this characteristic could be conferred by the glycosylation of the surface mannoproteins. Mannans present on the surface contribute to virulence by giving hydrophobicity, changing the adherence capabilities to the host cell, and suppressing the immune response [20]. Blastoconidia have a hydrophilic surface, but the formation of a germination tube is associated with the adhesion capabilities of the yeast. Mannoproteins bind red blood cells and induce hemolysis. The hyphae, unlike the blastoconidia, bind to hemoglobin to use it as a source of iron and for this reason have many more receptors for hemoglobin itself. The cell wall has three types of molecules for adhesion: (a) a glycoprotein belonging to the 2-integrin family, (b) a protein portion of a glycoprotein, and (c) a polysaccharide portion of a mannoprotein [21,22,23]. It is known that the protein fragment iC3b, which is part of the complement system component of the immune system, is produced when the complement factor I cleaves C3b. Complement receptors on white blood cells can bind iC3b and cannot bind to factor B, thus preventing amplification of the complement cascade via the alternative pathway. Furthermore, the complement factor I can split iC3b into C3d [21]. The glycoprotein binds the arginine-glycine-aspartate (RGD) sequence, which is common in many glycoprotein matrices of host organisms (it is expressed on the hyphae surface) acting as a receptor, thus mimicking complement iC3b and C3d. Finally, the polysaccharide portion of the mannoprotein binds a host receptor, which is currently still under study [24]. Important virulence factors are represented by enzymes which induce yeast to lysate the cell membrane, such as phospholipase, lipase, SAP (secreted aspartyl proteases), and hydrolytic and adhesion enzymes [22,23]. There are different isoforms of SAP (from SAP1 to SAP10) that produce different types of clinical effects (e.g., SAP1–3 causes superficial cutaneous or mucosal infections in experimental models, while SAP 4, 5, and 6 are important for systemic candidiasis). The production of phospholipase is essential for virulence because it models the lipid substrate of the membrane by means of the lipid hydrolysis of the host cell. Phospholipase B is produced during the infection process and is limited by pH changes [24,25]. Finally, another virulence factor would be the formation of biofilms that develop on the surfaces of objects (catheters, etc.). The formation of biofilms can occur in a first phase in which the cells adhere to the surface of the foreign body and form a layer of cells and increase the synthesis and production of hyphal wall proteins; then, an active cell growth occurs, an extracellular matrix is produced, and hyphae are formed which stabilize the biofilm. The hyphal is the final stage, as in some conditions it will remain as a yeast form due to the presence on quorum sensing molecules, such as farnesol, to allow the dissemination of *Candida albicans*. The biofilm provides protection to yeast cells against the host’s immune system and resistance to antifungal drugs [26,27,28].

### 2.2. The Genome

The genome is diploid with asexual replication. However, it has been detected that there exists a process activation of divergence, appointed with the term “(para)sexuality cycle”, thus without evidence of meiosis, and involving mating, recombination, and genomic reduction. This condition increases its ability to recombine and adapt to various environments, helping its growth and proliferation [29,30]. In fact, the CUG and CTG codons (that encode for leucine) in *Candida* spp. encode for serine. This led to the change in the serine tRNA loop, which could be the cause of its thermos tolerance [30,31]. *C. albicans* has eight chromosomes, numbered from 1 to 7, plus a highly variable chromosome called R. Previous molecular epidemiological studies have shown that a single group of genetically related *C. albicans* may predominate in a given patient population, that is the gene clad A. Thus, this widely represented genotypic cluster among *C. albicans* representing the so called general-purpose-genotype cluster (GPG). In a subsequent comparative study, the data showed that cluster A constitutes a generic, geographically widespread genotype and is a predominant etiological agent of all forms of candidiasis in the patients studied and belongs to the GPG [32,33]. The next step was to find out the correlation between GPG and the ALS7 gene (agglutinin-like sequence; protein with a key role in yeast adherence). The ALS7 gene was found to have two modulable regions [34]. The first contains sequences called tandem repeat domain and the second region contains VA/TSES sequences (of the coded amino acid sequence). Based on the analysis of these sequences, 49 genotypes of *C. albicans* were identified. The GPG genotype was found to be predominantly associated with strains that contained a high repeat count of both tandem and VA/TSES. Many other genes and open reading frames related to the GPG genotype, such as FUR1, YHB4, and YWP1, were subsequently identified. In a study in neonatal intensive care units, the mortality rate was 45.5% among infants infected with the GPG + strain [35,36].

### 2.3. The Immune Response

Candida can turn into a pathogen when the immune system is compromised. All components of the immune system are involved in the recognition and thus in the defense against C. albicans. In healthy subjects, innate immunity reacts by identifying yeast cells, and cell-mediated immunity and cytokines have the task of protecting the mucous membranes, while the role of humoral immunity is not entirely clear [37]. Monocytes on their membrane have receptors called toll-like receptor (TLR), and to a lesser extent by neutrophils, which recognize specific sites of the membrane structure of C. albicans antigen, expressing high concentrations of pattern recognition receptors (PRR), which are also used for the recognition of the fungi. The neutrophils, in addition to the TLRs, show a strong expression of complement receptor 3 (CR3) and of receptors that bind the Fc region of immunoglobulins (Fcγ) [37,38]. Membrane proteins, such as mannans and mannoproteins, that increase cytokines are recognized. Those in turn inhibit in a non-specific way the immunity mediated in response to the antigen, as well as disadvantaging the maturation of dendritic cells [39]. The various receptors bind to the PAMP regions (pathogen-associated molecular patterns), leading to the production of cytokines, and to the phagocytosis of the fungus. In vitro evidence reported of how the glucans, released following a deep fungal infection, stimulate the production of leukocytes and pro-inflammatory factors, such as cytokines and chemokines’ molecular patterns that lead to a series of metabolic processes and enzymatic cascades that also stimulate the production of cytokines, to the phagocytosis of the fungi. Following the opsonization, the yeast is eliminated through oxidative and non-oxidative mechanisms present in the cell [40,41]. Finally, thanks to an interaction between commensal and/or environmental microorganisms and the immune system, a tolerance and “good neighbor” status (eubiosis) occurs in normal healthy individuals. To make it all happen, some commensal fungi have invented tolerance mechanisms for the host’s immune system in order to establish colonization. It has been noted that there is a high concentration of specific regulatory T cells (for *C. albicans*) in the blood and that is five to ten times higher than the specific effector T cells for the same fungus. It was noted in mice that the indoleamine 2,3-dioxygenase of the fungi led to the development of tolerogenic dendritic cells of the intestinal Peyer’s patch. Hence, this leads to the degradation of tryptophan, which will activate the hydrocarbon receptor aryl (AhR) in T cells to thus promote tolerant regulatory T cells [40,41].

## 3. The Role of Oral Dysbiosis

In healthy individuals, the *Candida* spp. is found in mucous membranes, such as the gastrointestinal tract, mouth, nose, reproductive organs, skin, etc. It is a member of the resident human microbiota. Bacteria, virus, and fungi colonize all parts of the mouth, and are components of the permanent microorganisms that connect and communicate with each other creating a biomembrane. They affect their host in many ways and are responsible for diseases in the oral cavity or other parts of the body (Table 1) [42,43].

Traditional cultivation methods isolate only 60% of oral bacteria. The culture of oral material provides a variety of microorganisms, including those that are necessarily aerobic and anaerobic. These organisms have a wide range of metabolic capabilities, including the ability to reconstruct sugars and proteins and complex substrates found in the oral cavity [44]. Today, one of the most important databases about the oral human microbiome is the Human Oral Microbiome Database (eHOMD). The *Streptococcus* predominates in the oral cavity and *S. hemolytic A* is more often isolated (presence rates are: 28% dental plaque, 46% saliva, 45% tongue, and 29% gingival cleft) and fungi as part of the oral microbiome make up a small percentage. The predominant species is *C. albicans*, and other fungal genera in the oral cavity are *Cladosporium, Saccharomyces*, *Aspergillus*, *Fusarium*, and *Cryptococcus*. Several studies suggest that the dysbiosis that leads to an overpopulation of *Candida* spp. increases the risk to malignant cell transformation. Indeed, in oral neoplasms, there is a deterioration of the composition of the fungi with an over-representation of the genera *C. albicans*, *Hannaella*, and *Gibberella* in oral neoplasms [42,43,44]. In this case, it is possible that the profile of the oral microbiome can function as a diagnostic biomarker in the prevention or diagnosis of those malignant diseases, such as the oral cancer’s one. The germs of normal flora interact with each other and with their host through complex networks. Usually, these interactions lead to a “harmonious” coexistence (eubiosis) and when balance is disturbed (dysbiosis), various diseases may develop [42,45,46,47,48,49]. The oral microbiome plays an essential role in maintaining health in the oral cavity and in the general physiology of the human body. It acts as a barrier to the installation of other germs, some of which may be potentially pathogenic as they help increase the natural and acquired immunity of the host. This is due to the interactions between microbes and between microbes and host. The presence of microorganisms such as *Streptococcus salivarius* K12 (secretes a bactericidal inhibitor that inhibits the growth of Gram-negative species associated with periodontitis and halitosis) and others in the normal microflora of the mouth inhibits the colonization by other germs, some of which may be pathogenic [50,51]. Indeed, the use of bacterium therapy with probiotics helps immune defenses and in the concept of eubiosis of the microbiota [51,52]. The consumption of large amounts of carbohydrates causes the production of acids that the saliva no longer could regulate, leading to a continuous pH decrease. This in turn changes the composition of the microbiome in the mouth because it favors microorganisms that thrive in acidic pH [53].

*C. albicans* is the only yeast of clinical relevance to have different morphological aspects, such as blastoconidia, elongated pseudohyphae, real hyphae, and in special conditions, chlamydospores. *Candida* spp. cause a wide range of acute and chronic infections, especially in patients with weakened immune systems, and thus can become pathogenic under specific conditions (opportunism) causing candidiasis [20,21,22,23]. Through the intestine, it can reach the blood where it releases its toxins, causing candidemia with symptoms of abdominal swelling, slowing of digestion, intestinal disorders (constipation or diarrhea), food intolerances, fatigue, irritability, insomnia, memory loss, headache, and depression [15,28]. Oral candidiasis is a common occasional fungal infection of the oral mucosa caused by excessive growth of *Candida* species, with the most common species being *C. albicans* [17,37]. Factors contributing to its onset include dysfunction of the salivary glands, certain medications, artificial prosthetics, and a high-carbohydrate diet, while cigarette smoking, diabetes, cancer, and immunosuppression appear to be of particular importance in oral candidiasis. The treatment of excessive growth of *Candida* does not seek to eliminate *C. albicans* from the individual, but to restore its correct and balanced ecological relationship between human and microorganisms. In the saliva, there are various antimicrobial substances, such as lactoferrin, amylase, glycosylated proline-rich protein (PRP), lysozyme, and special antibodies against *Candida*, interacting with the balance of the mucous membranes and controlling oral candidiasis and preventing excessive growth in its population. Reduced functionality of the salivary glands can cause oral candidiasis [15,54,55]. Many medications, such as inhalation steroids due to the suppression of cellular immunity and phagocytosis, have shown to increase the risk of oral candidiasis. The local immunity of the mucosa is restored to normal after the termination of the steroids by inhalation. Broad-spectrum antibiotics destroy the local oral flora in favor of *Candida* proliferation. Additional dental components contribute to *Candida* infection because they create a microenvironment conducive to its development with anaerobic and low pH, and *Candida’s* growth in saliva and its attachment to oral epithelial cells is boosted by a carbohydrate-rich diet [28,43,54,55]. There was also a significant percentage of other fungi, such as *Aspergillus*, *Penicillium*, *Schizophyllum*, *Rhodotorula*, and *Gibberella*. Scientists eventually failed to find a significant difference in the overall composition of fungi between periodontally affected and healthy areas, although there was a clear correlation between caries, tooth loss due to to the apoptotic effect induced by TNF-related apoptosis-inducing ligand (TRAIL), and increased prevalence of *Candida* spp. [56,57].

**Figure 1:**
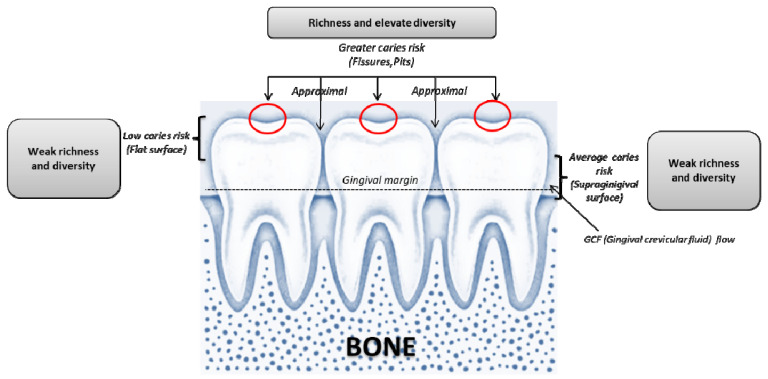
The risk associated with the areas that may be affected by caries and periodontal disease. Surfaces and locations with the highest variety and richness of oral microbial communities are more sensitive to caries and in the genital areas of periodontal disease. When tooth decay or periodontitis develops, the acidic environment reduces the variety and richness of local microbes.

Interactions have recently been observed between *C. albicans* and *Porphyromonas gingivalis*, which have shown that cohesion caused by specific proteins causes significant changes in gene expression by *P. gingivalis*, which could be used to increase infectivity. This means that interactions between different groups of microorganisms can trigger specific oral diseases such as the precancerous conditions. All the above show that the etiological flora of periodontal disease is not yet revealed and that several fungi can be a parameter responsible for the onset and progression of periodontal infection (Figure 1) [58,59].

## 4. The Current Clinical Evidence about *C. albicans’* Role in Oral Cancer

### 4.1. Epidemiological Findings

The genus *Candida* has about 200 species, of which twenty are most often isolated during infection, such as *C. albicans* and *non-albicans Candida* (e.g., *C. tropicalis*, *C. glabrata*, *C. parapsilosis*, *C. krusei*, *C. guilliermondii,* and others). However, the *C. albicans* species is the most common found and the frequency of non-*C. albicans* species is constantly increasing, while in the study of single patient groups, a slight variation in the distribution of these species is observed [60]. It has been noted that unlike adults, pediatric patients are more likely to develop candidiasis than *C. albicans* and *C. parapsilosis*. Several epidemic cases have been reported, especially in high-risk wards, such as geriatric, hematology, centers for the treatment of severe burns, intensive care units (ICU), and transplantation units. Newborns are often contaminated at the time of delivery by *Candida* present in the mother’s vagina (particularly common during the third trimester of pregnancy and over 56% of women in labor are affected) [60,61]. *Candida* bloodstream infection and deep-seated candidiasis may lead to a severe septic state and have a mortality that can range up to a maximum of 50% [62].

The presence of oral candidiasis (with an incidence from 7% to 52%) has been observed in patients with neoplastic pathology (such as head and neck malignancies, hematopoietic neoplasms, and others) and undergoing chemotherapy and/or radiotherapy [63,64]. Indeed, in a study with 100 patients who were affected by oral cavity squamous cell carcinoma, the presence of *C. albicans* was found with an incidence of 84% compared to the other species of fungi found [65,66]. In a 25-year observational study following 520 cases of oral leukoplakia, it was concluded that there was a link between *Candida*-infected oral leukoplakia and malignant neoplasms. *Candida* infection presented in 13.5% of oral leukoplakia cases and the malignant transformation rate of these was 28.7% (20 out of 70 patients) [67,68]. Another study showed an association between *Candida* and malignant transformation of oral leukoplakia of 257 patients. Cultures showed that 31% of patients were positive for *Candida* and two of these patients subsequently developed carcinoma with a malignant transformation rate of 2.5% [69].

In a case-control biopsy study of 78 patients with oral leukoplakia, the results showed that *Candida* infection was present for 39.7%, and 28 of these patients showed dysplasia (90.3%: 4 cases with mild dysplasia, 14 with moderate dysplasia, and 10 with severe dysplasia). Afterwards, two cases had a malignant transformation [70]. In a preliminary study (in vitro), chronic hyperplastic candidiasis or leukoplakia with *Candida* super infection can significantly promote cancer. However, the presence of oral *Candida* can be a sign of immunodeficiency and therefore it is important to investigate [71]. In several studies, it has been noted that the presence of *Candida* in oral squamous cell carcinomas is associated with a worse prognosis and the incidence rate of *Candida* infection reported in oral squamous cell carcinoma of the head and neck region varies from 25% to 74.7% [65,72].

### 4.2. C. albicans and Oral Malignant Transformation Condition

*C. albicans* infection can contribute to the carcinogenesis process like known carcinogenic substances even in animal models. In some cases of dysplasia there is a significant association between candidiasis and the increasing degree of dysplasia in oral potentially precancerous disorders, and between oral squamous cell carcinoma and oral potentially precancerous disorders, but there is no significant correlation in epithelial dysplasia in mucous membranes affected by oral potentially precancerous disorders [73,74,75,76,77,78,79,80]. In some case reports with oral leukoplakia, *C. albicans* infection is significantly associated and furthermore this association is more marked if the oral leukoplakia is localized to the tongue, and in dysplastic and neoplastic forms of oral leukoplakia. In a systematic review conducted on the influence of *Candida* infection and leukoplakia on a malignant transformation, it showed that *Candida* may play a role in dysplastic and malignant transformations [75]. Finally, the formation of invasive hyphae of *C. albicans* occurs based on the production of IL1β, which activates the production of proinflammatory cytokines. The molecular investigation data have also identified, in *C. albicans* genotype A, significant colonization in oral squamous cell carcinoma lesions, postulating that genotypic diversity may affect the carcinogenic process [76,77,78,79,80].

## 5. The Biomolecular Mechanisms of *C. albicans*-Induced Oncogenesis

**Figure 2:**
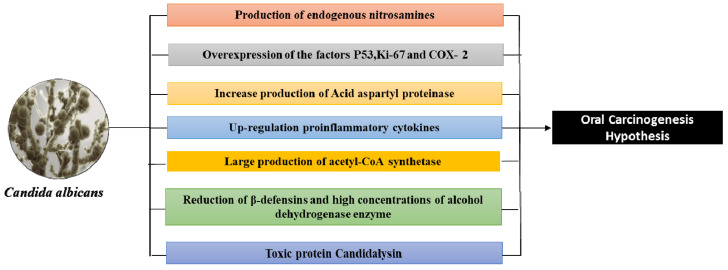
The main hypothetical molecular mechanisms by which *C. albicans* can cause precancerous and malignant neoformations in the oral cavity.

There are several hypothetical molecular mechanisms, that according to each *Candida*, can cause dysplasia and malignant neoformations in the oral epithelium and can be mainly from [43,54,55,56,57,58]:

- The production and release (via hyphal invasion) of nitrosamines, such as *N*-nitroso-benzyl-methylamine (e.g., caused by dysbiosis of the oral microbiota), which can lead to a tumor condition in mouse models (such as the Sprague-Dawley rats) [80,81,82,83].
- An over-expression of P53, *Ki*-*67 labeling index*, and *Prostaglandin*-*endoperoxide synthase* 2 (COX-2) are some of the additional mechanisms by which *Candida* can affect malignant transformation into oral leukoplakia. P53 and Ki-67, which are markers of cell proliferation, have overexpression that is well established in malignant lesions, and COX-2, which is markedly increased in inflammation states and is associated with the release of prostaglandins, thus influencing cell proliferation, cell death, and tumor invasion [67,84,85,86].
- *Acid aspartyl proteinase* appears to be more present in oral lesions and therefore also in those with leukoplakia than in healthy subjects [67]. The production of acid aspartyl-proteinase are putative virulence factors in candidiasis, and are why an acidic pH exists, thus degrading the sub endothelial extracellular matrix, as well as laminin 332 and E-cadherin. This induces dysplastic alterations and thus begins the *C. albicans* dissemination in the systemic circulation and therefore in the organs [48,85,86]. On the other hand, in a model of hyphal invasion (localized or uniform) of *Candida*, there is no difference between oral potentially precancerous disorders and oral squamous cell carcinoma. These biomolecular mechanisms highlight the ability of *Candida* to influence malignant and cellular changes in oral leukoplakia [86].
- Oral *Candida* infection is a cause of *up-regulation in proinflammatory cytokines* (interleukin (IL)-1α, IL-1β, IL-6, IL-8, IL-18, tumor necrosis factor (TNF)-α, IFN-γ, and GM-CSF), that influences the metabolic pathways and induces directly an endothelial dysfunction, playing a role in immune-related mechanisms with cancer development [48,62,63].
- *C. albicans* can produce acetaldehyde (carcinogen due to mutagenic qualities in DNA) from precursors found in the oral cavity (metabolizing ethanol and glucose in high quantities, especially when associated with smoking and alcohol consumption) [67,72,84]. Thus, *Candida* can produce large quantities of acetaldehyde and acetyl-CoA synthetase (more in smokers) in cases of potentially malignant disorders and in oral carcinomas (concentrations of acetaldehyde and acetyl-CoA synthetase increase) compared to healthy individuals and those with ectodermal dystrophy and autoimmune polyendocrinopathy (with candidiasis) [72,85,86,87,88,89,90,91]. However, the increase in the mutagenic amounts of acetaldehyde is more marked even in occupationally exposed workers to carcinogen [92] and people with poor oral hygiene, than in healthy subjects, via the oral microbiota (*Streptococcus viridans* and resident fungi such as *Candida*) that can convert ethanol into acetaldehyde (possess the enzyme alcohol-dehydrogenase) [93]. Indeed, the levels of acetaldehyde produced by *Candida* increase in proportion to the increase in alcohol consumption [93,94].
- In oral squamous cell carcinoma, the reduction of β-defensins favors *Candida* superinfections. In chronic hyperplastic candidiasis, C. *albicans* is the predominant species and is associated with high concentrations of alcohol dehydrogenase enzyme and P53 that suggests a dysplastic potential factor [95]. In fact, there is evidence that *Candida’s* epithelial invasion can cause hyperplastic conditions (Figure 2) [67,72,96].
- The candidalysin (or 31-amino acid α-helical amphipathic peptide) is a cytolytic toxin of *C. albicans*. It is encoded by the ECE1 gene initially associated with fungal filamentation ability (release the toxin from the hypha) and host cell adhesion. Initially, ECE1 encodes 271 amino acid pre-proproteins that are cleaved by Kex8p enzyme into eight smaller peptides (Ece1-I to Ece1- VIII). Ece1-III6-93 is an epithelial immune activator and collaborates with the cytolytic activity of *C. albicans* [97]. Likewise, candidalysin is an inducer for NF-κB and MAPK pathways. Candidalysin has been reported to excite granulocyte macrophage colony-stimulating factor GM-CSF, an essential molecule in carcinogenesis. After the macrophage death, the *C. albicans* can escape, survive, and outgrow other macrophages. On the other hand, it induces epithelial damage and elicits host inflammatory processes because it is a trigger for NLR family pyrin domain containing protein 3 (NLRP3) [98].

## 6. Conclusions

From the literature, it would appear that *C. albicans* plays an important role in the risk for precancerous lesions of the oral mucosa as well as for moderate and severe dysplasia (in the dysplastic and malignant transformation of oral lesions). Furthermore, the ability of *Candida* to produce carcinogens such as nitrosamine and acetaldehyde, and the induction of proinflammatory cytokines, can be risk factors in the development of oral cancer. In fact, the potential role of yeast in oncogenic processes in the oral cavity remains the subject of much debate. There is evidence that *Candida* is the direct cause of oral cancer probably only when the infection is chronic, deep, and associated with risk factors such as tobacco and alcohol, or others.

## Figures and Tables

**Table 1:** The main diseases associated with dysbiosis of the human microbiota.

Oral Microbiome and Associated Diseases	
Oral	Systemic
Caries Periodontal (gingival, periodontitis) Peri-implantitis Infections (candidiasis) Mucosal lesions (leukoplakia, lichen planus, local systemic lupus erythematosus) Cancer	Gastrointestinal system (IBD, cirrhosis, cancer) Nervous system (Alzheimer’s) Endocrine system (diabetes, polycystic ovary syndrome, adverse pregnancy outcomes, obesity) Immune system (rheumatoid arthritis, human immunodeficiency virus infection) Cardiovascular system (atherosclerosis, endocarditis)

## Data Availability

Not applicable.
